# Dr. Arthur's Book

**Published:** 1872-12

**Authors:** Jos. H. Scales

**Affiliations:** VA.


### ARTICLE IV.

### *Dr. Arthur's Book.*

BY JOS. H. SCALES, D. D. S., OF VA.

Although an humble, I claim to be an honest member of our profession; and as such, enter my protest against the dogmatism of the late work of Dr. Arthur, of Baltimore. While I would not say ought in disparagement of the distinguished author, whose best energies have been given to the advancement and elevation of dentistry; although he is a *patriarch*, and I only an *infant*, comparatively speaking, I respectfully demur to the general tone pervading it. He may be correct in the main, yet, I think it presumptuous in any man to lay down so dogmatically his experience and observations as rules for the guidance of fourteen thousand others; no matter what his age or experience may be. Every dentist should cull from the experience of "the fathers;" exercising his own brain to the utmost, so as to render his practice, as far as possible, an eclectic one; but, at the same time, should carefully guard against adopting the vagaries and idiosyncrasies of enthusiasts. 'Tis for the benefit of the masses of mankind we are laboring; and in order to accomplish the most good, must necessarily avail ourselves of every means to attain this result. Every thinking and observing man must admit that we have been derelict in our duty, in not educating the masses as to the importance and proper mode of preserving the teeth. Yet, I think it unfortunate for Dr. A. and his book, that it should have been addressed, both to the profession and people; more especially, since he confines them to "the learned few," or those who "think so too" with him, in electing who shall treat their own or families' teeth. Is it possible that our science is retrogressive, instead of progressive, after twenty years toilsome, diligent, and painstaking investigation? Such must be the conclusion of the reader who accepts Dr. A's *ipse dixit.* Much of our practice might be stigmatized by "the learned few" as quackery; yet we do the best we can under the circumstances. We can no more promise to save every tooth we treat, than a physician can to save the life of every patient he is called to see. Temperament, constitutional peculiarities, faithful following prescriptions, and many other conditions, are as essential to success in the one as the other.

---

